# Effect of dapagliflozin on obstructive sleep apnea in patients with type 2 diabetes: a preliminary study

**DOI:** 10.1038/s41387-019-0098-5

**Published:** 2019-11-04

**Authors:** Yi Tang, Qin Sun, Xiao-Yan Bai, Yun-Fan Zhou, Qiong-Lan Zhou, Min Zhang

**Affiliations:** 1 Department of Endocrinology, The Fifth People’s Hospital of Chengdu, 611130 Chengdu, P.R. China; 2 Center of Diabetes Mellitus, Sichuan Academy of Medical Sciences and Sichuan Provincial People’s Hospital, 610072 Chengdu, P. R. China; 3 Department of Respiratory, The Fifth People’s Hospital of Chengdu, 611130 Chengdu, P.R. China; 4 Department of Endocrinology, The Third People’s Hospital of Chengdu, 610000 Chengdu, P.R. China; 5 Department of Endocrinology, People’s Hospital of Yilong County, 637676 Yilong, P.R. China

**Keywords:** Type 2 diabetes, Diagnostics

## Abstract

**Objective:**

The aim of this case-control study was to assess the efficacy of dapagliflozin combined with metformin for type-2 diabetes mellitus (T2DM) with obstructive sleep apnea hypopnea syndrome (OSAHS).

**Methods:**

A total of 36 patients with newly-diagnosed T2DM and OSAHS were randomized divided into two groups. Eighteen OSAHS patients with T2DM, who were treated with dapagliflozin and metformin, were assigned as the dapagliflozin group. These patients were given dapagliflozin and metformin for 24 weeks between February 2017 and February 2018. Another 18 OSAHS patients with T2DM, who were treated with glimepiride and metformin for 24 weeks, were assigned as the control group. Fasting plasma glucose (FPG) level, postprandial blood glucose (PPG), hemoglobin A1C (HbA1c), fasting insulin, homeostasis model assessment of insulin resistance (HOMA-IR), blood lipids, body mass index (BMI), blood pressure, apnea-hypopnea index (AHI), minimum oxygen saturation (LSpO_2_), and Epworth Somnolence Scale (ESS) score were measured before and at 24 weeks after the initiation of treatment.

**Results:**

In the dapagliflozin group, triglyceride (TG), systolic pressure (SBP) and diastolic pressure (DBP) significantly decreased following treatment, while high-density lipoprotein cholesterol (HDL-C) significantly increased (*P* < 0.05). Furthermore, a reduction in AHI, an increase in LSpO_2_ and a decrease in ESS score were observed in the dapagliflozin group (*P* < 0.05), but not in the control group. Moreover, blood glucose, HbA1c, HOMA-IR, and BMI significantly decreased in these two groups, and the decrease was more significant in the dapagliflozin group.

**Conclusion:**

These present results indicate that dapagliflozin can significantly reduce glucose, BMI, blood pressure and AHI, and improve hypoxemia during sleep and excessive daytime sleepiness, which thereby has potential as an effective treatment approach for OSAHS.

## Introduction

Obstructive sleep apnea hypopnea syndrome (OSAHS) includes a constellation of symptoms characterized by total obstruction (apnea) or partial obstruction (hypopnea) of the upper airways during sleep^1^. Patients with OSAHS have apnea or reduced breathing during sleep, which causes anoxia, carbon dioxide retention, nocturnal sleep apnea, and excessive daytime sleepiness (EDS). Furthermore, OSAHS can induce cognitive decline and autonomic modulation disorder. In addition, OSAHS is closely correlated to obesity, type-2 diabetes mellitus (T2DM) and cardiovascular diseases^2,3^, and 60–70% of OSAHS patients are overweight or obese. The prevalence of OSAHS in the T2DM patients with obesity is 86%^4^. Untreated moderate and severe OSAHS is considered to be a key risk factor for cardiovascular events and death^5–7^. Many patients suffer from obesity. Clinical practice guidelines recommend that overweight adult OSAHS patients should reduce weight, because weight loss helps improve ventilation and prognosis in these patients^8,9^.

A new hypoglycemic drug, dapagliflozin, which is a kind of sodium glucose co-transporter 2 (SGLT2) inhibitor, can reduce the reabsorption of glucose by the kidney, increases urine glucose excretion, and reduce blood glucose levels in T2DM patients^10,11^. It has also been found that this inhibitor can reduce weight, lower blood pressure, and improve hyperlipidemia^12–14^. Another classic hypoglycemic drug, metformin, has been proven to benefit weight loss in T2DM patients^15,16^.

Dapagliflozin and metformin can effectively reduce the body weight of patients with OSAHS^17^. Therefore, it is assumed that these might have potential effects in improving the symptoms, and even the breathing of OSAHS patients. In order to verify this idea, a randomized-controlled trial was designed to observe the effects of metformin combined with dapagliflozin on body mass index (BMI), and the breathing and sleep quality of patients with OSAHS combined with T2DM.

## Materials and methods

### Patients

A total of 36 patients with newly diagnosed T2DM and OSAHS were included in the study cohort. These patients were enrolled from the Fifth People’s Hospital of Chengdu and Sichuan Provincial People’s Hospital between February 2017 and February 2018. The diagnose of OSAHS was completed based on symptoms, such as snoring, respiratory arrest, suffocation, daytime drowsiness, fatigue, lack of energy and the relevant inspection. The age of these participants ranged within 18–65 years old, and their HbA_1C_ was >7.5%, but <9%. All these participants could not effectively control their blood glucose when using the largest dose of metformin, and needed other hypoglycemic drugs. Patients with moderate–severe kidney failure and liver damage were excluded to decrease the onset of oral antidiabetic drug-related side effects. Patients with serious lung function decline, neuromuscular diseases, and congestive heart failure, as well as patients who recently had acute infection and surgery, were also excluded. All patients were recommended to adopt the routine diet and exercise regimen for patients with diabetes.

A total of 36 patients were divided into two groups: dapagliflozin group (*n* = 18), and control group (*n* = 18). The randomization was performed by a computer-generated number. Allocation was issued using opaque, sealed, numbered envelopes. The sample size was calculated by the statistical software. The dose of the hypoglycemic drug was regulated according to the blood sugar in the whole trial. These two groups were required to receive a large dose of metformin (0.85 g bid). All 18 patients in the dapagliflozin group received 5 mg of dapagliflozin every day, and 10 mg was added one week later, for a total of 24 weeks. The 18 subjects in the control group received 2 mg of glimepiride every day. If the glucose of these patients were not well-managed, this could be increased to 4 mg/day. The treatment duration also lasted for 24 weeks.

The present study was approved by the ethics committees of the Fifth People’s Hospital of Chengdu and Sichuan Provincial People’s Hospital, and both committees provided a waiver of consent. All participants had signed the informed consent.

### Evaluations

The primary outcome was glucose level, including fasting plasma glucose (FPG) level, and postprandial blood glucose (PPG). Besides, hemoglobin A1C (HbA1c), fasting insulin, homeostasis model assessment of insulin resistance (HOMA-IR), blood lipids, BMI, blood pressure, apnea–hypopnea index (AHI), and minimum oxygen saturation (LSpO_2_) were also detected, and the severity of daytime sleepiness was evaluated using the Epworth Somnolence Scale (ESS) score. Blood glucose and HbA1c was respectively determined using the glucose oxidase method and high performance liquid chromatography. FPG, PPG, HbA1c, fasting insulin and blood lipids were detected by ANAYTECH-640 automatic biochemical analysis. AHI and LSpO_2_ were measured using a portable-type sleep apnea detector (JF-1000, Beijing YiheJiaye Medical Technology Corporation). The above indexes were reviewed after 24 weeks.

### Statistical analysis

SPSS 23.0 statistical software was used to statistically analyze the clinical data. Measurement data were expressed with mean ± standard deviation. The normally distributed continuous data were compared using independent *t*-test, while the non-normally distributed continuous data were compared using non-parametric tests. The count data were analyzed using chi-square test. If *P* < 0.05, the difference between groups was considered to be statistically significant.

## Results

### Patient characteristics

Table 1 presents the characteristics of the 36 patients with OSAHS and T2DM before the follow-up. There were no significant differences in clinical profiles between the dapagliflozin group and control group.

**Table 1:** Clinical characteristics at start of follow up

	Dapagliflozin (*n* = 18)	Control (*n* = 18)	*P*-vaule
Age	56.10 ± 7.21	57.88 ± 10.07	0.813
Male (%)	55%	65%	0.519
History	1.12 ± 0.30	1.15 ± 0.26	0.742
BMI (kg/m^2^)	28.17 ± 1.21	28.04 ± 1.19	0.746
FPG (mmol/L)	8.24 ± 0.29	8.12 ± 0.47	0.360
PPG (mmol/L)	14.86 ± 1.58	14.76 ± 1.63	0.861
HbA1c (%)	8.38 ± 0.95	8.45 ± 1.10	0.830
HOMR-IR	4.03 ± 0.31	4.06 ± 0.33	0.786
TG (mmol/L)	1.85 ± 0.47	1.89 ± 0.40	0.824
LDL (mmol/L)	2.79 ± 0.32	2.67 ± 0.49	0.907
HDL (mmol/L)	0.96 ± 0.13	0.95 ± 0.14	0.941
SBP (mmHg)	151.22 ± 5.95	152.56 ± 7.25	0.551
DBP (mmHg)	92.44 ± 7.62	93.16 ± 6.23	0.887
AHI(events/h)	37.45 ± 6.04	38.11 ± 6.27	0.747
LSp O2 (%)	84.06 ± 14.58	83.72 ± 13.77	0.812
ESS score	11.83 ± 3.76	12.50 ± 3.79	0.600

*TG* Triglyceride, *LDL* low density lipoprotein cholesterol, *HDL* high density lipoprotein cholesterol, *SBP* systolic pressure, *DBP* diastolic pressure

### Changes in clinical and laboratory findings

The changes in clinical and laboratory findings for the dapagliflozin and control groups are presented in Table 2. After 24 weeks of treatment, FPG, PPG, HbA1c, HOMA-IR, and BMI significantly decreased in these two groups (*P* < 0.05). Compared with the control group, HOMA-IR and BMI significantly decreased in the dapagliflozin group (*P* < 0.05), while FPG, PPG, and HbA1c did not decrease (*P* > 0.05). The glucose levels in these two groups were all effectively managed. Compared to the control group, and although the regimen of dapagliflozin combined with metformin had no significant advantage in reducing glucose, the treatment scheme could be more effective in improving the patient’s insulin resistance, and reducing the patient’s weight.

**Table 2:** Changes of clinical and laboratory findings after treatment

	Dapagliflozin (*n* = 18)			Controls (*n* = 18)			Difference	
	Baseline	Posttreatment	*P*-vaule	Baseline	Posttreatment	*P*-vaule	Mean	*P*-vaule
BMI (kg/m^2^)	28.17 ± 1.21	25.92 ± 0.92	0.000	28.04 ± 1.19	27.04 ± 1.11	0.000	−1.21 ± 0.85	0.004
FPG (mmol/L)	8.24 ± 0.29	6.56 ± 0.38	0.000	8.12 ± 0.47	6.53 ± 0.32	0.000	0.02 ± 0.28	0.850
PPG (mmol/L)	14.86 ± 1.58	9.95 ± 1.62	0.000	14.76 ± 1.63	10.23 ± 0.95	0.000	−0.21 ± 1.40	0.563
HbA1c (%)	8.38 ± 0.95	6.49 ± 0.71	0.000	8.45 ± 1.10	6.35 ± 0.36	0.000	0.18 ± 0.75	0.473
HOMR-IR	4.03 ± 0.31	3.07 ± 0.27	0.000	4.06 ± 0.33	3.94 ± 0.25	0.010	−0.79 ± 0.49	0.000
TG (mmol/L)	1.85 ± 0.47	1.58 ± 0.51	0.004	1.89 ± 0.40	1.86 ± 0.38	0.220	−0.28 ± 0.31	0.028
LDL (mmol/L)	2.79 ± 0.32	2.77 ± 0.28	0.174	2.67 ± 0.49	2.61 ± 0.34	0.301	0.15 ± 0.51	0.172
HDL (mmol/L)	0.96 ± 0.13	1.06 ± 0.12	0.001	0.95 ± 0.14	0.96 ± 0.13	0.084	0.09 ± 0.14	0.036
SBP (mmHg)	151.22 ± 5.95	146.11 ± 6.45	0.000	152.56 ± 7.25	150.25 ± 7.38	0.725	−6.11 ± 6.63	0.012
DBP (mmHg)	92.44 ± 7.62	86.33 ± 7.17	0.000	93.16 ± 6.23	92.12 ± 7.16	0.907	−5.88 ± 8.56	0.019
AHI (events/h)	37.45 ± 6.04	26.72 ± 4.69	0.000	38.11 ± 6.27	36.1 ± 4.50	0.265	−10.17 ± 6.96	0.000
LSp O2 (%)	84.06 ± 14.58	87.16 ± 13.56	0.004	83.72 ± 13.77	84.12 ± 13.83	0.479	3.00 ± 4.46	0.020
ESSscore	11.83 ± 3.76	9.05 ± 2.75	0.001	12.50 ± 3.79	12.11 ± 3.16	0.422	−1.78 ± 2.16	0.004

*TG* triglyceride, *LDL* low density lipoprotein cholesterol, *HDL* high density lipoprotein cholesterol, *SBP* systolic pressure, *DBP* diastolic pressure

In the dapagliflozin group, TG, SBP, and DBP significantly decreased after the treatment, while HDL-C significantly increased (*P* < 0.05). The significant changes of these characteristics were not observed in the control group.

A reduction in AHI, an increase in LSpO_2_, and a decrease in ESS score were observed in the dapagliflozin group (*P* < 0.05), but not in the control group, indicating the amelioration of breathing and EDS after the administration of dapagliflozin and metformin.

## Discussion

The present study found that the combination of dapagliflozin and metformin not only alleviated the patient’s weight, and improved the blood glucose, blood pressure, and blood lipid levels of patients with T2DM and OSAHS, but also significantly improved the patient’s ventilation and daytime sleepiness, showing that the symptoms of OSAHS patients were ameliorated. Previous studies have pointed out that metformin can reduce the weight of T2DM and OSAHS patients, but it did not enhance the ventilatory function^18^ in these patients, and did not appear to be able to prevent the development process^19^. Therefore, it was considered that dapagliflozin may have played a major role in improving ventilation in patients with T2DM and OSAHS.

Although the improvement of OSAHS is closely correlated to the degree of weight loss, the mechanism for improvement in AHI and LSpO_2_ by dapagliflozin in the present study remains unclear. Weight loss maybe one reason, but the change in body composition may have been more important than the reduction in BMI^20–22^. Several researches have suggested that diuretic therapy can cause a decrease in AHI in patients with OSAHS, without a marked reduction in body weight^23–26^. Furthermore, researchers have inferred that diuretics lead to an overnight fluid shift from the legs to the neck during sleep^27^, with this redistribution partly contributes to the severity of obstructive sleep apnea. Moreover, dapagliflozin has diuretic effects, and changes the body composition^28,29^.

EDS is an important clinical feature of OSAHS. EDS is closely correlated to cognitive decline, anxiety, and depression. Economou et al. ^30^ examined 36 patients, and revealed that OSAHS patients with higher ESS scores have higher Hospital Anxiety and Depression Scale (HADS) scores, and that these patients had improved ESS and HADS scores after effective continuous positive airway pressure (CPAP) therapy. In addition, other studies have observed the remission of EDS and the relief in tension and stress of OSAHS patients after reducing their breathing disorder during sleep after OSAHS surgery^31–34^.

Untreated patients with severe OSAHS have more fatal and nonfatal cardiovascular events, when compared to patients with mild or moderate OSAHS^8,35^. Obesity and hyperglycemia would further increase adverse cardiovascular outcomes in patients with OSAHS^36^. Therefore, weight loss and blood glucose lowering are crucial for OSAHS adults with excessive weight and hyperglycemia, because obesity is closely associated with increased risk for OSAHS^8,37^, and weight loss may reduce OSAHS symptoms in addition to many other health benefits^38^.

CPAP and OSAHS surgery is recommended for adults with OSAHS. CPAP is the most effective treatment for OSAS, and improves the prognosis of patients with the disorder^8^. However, CPAP does not provide a permanent resolution^39^, and may not be well-tolerated by some patients, leading to poor compliance^40^. Surgical treatments, including uvulopalatopharyngoplasty, are associated with risks and serious adverse effects, and present evidence is limited, and is not able to demonstrate the benefits of surgery as a treatment for OSAHS^41^. Finally, pharmacologic therapies have not been part of the primary treatment, and no clinically useful medication is presently listed in OSAHS treatment guidelines.

If dapagliflozin can be widely confirmed to reduce OSAHS symptoms, it may be a more acceptable treatment, when compared to CPAP or surgery. It has the potential to not only treat T2DM, but also play a significant role in patients with severe OSAHS, thereby reducing the development of cardiovascular or cerebrovascular illnesses, and the risk of death.

*Limitation*: Firstly, the sample size was limited and a larger sample size was needed for further study. Secondly, the influence of dapagliflozin on neck circumference and other obesity-related signs remains unknown and should be further research. Thirdly, the relationship between neck circumference and AHI, LSpO_2_, ESS score were unclear and should be further investigated.

In conclusion, the investigators consider that dapagliflozin has therapeutic value for patients with T2DM combined with OSAHS. However, there are some limitations in the present study. (1) The study duration was short, and the cardiovascular events or deaths were not considered as the key points. Therefore, the present study was not able to determine whether dapagliflozin could improve the cardiovascular prognosis of these subjects. (2) The different effects between dapagliflozin and traditional treatment were not compared. Hence, more studies are needed in the future. On the one hand, large prospective randomized clinical trials are required to evaluate the effectiveness and safety of dapagliflozin in treating OSAHS, determine the additional health benefits that can be measured in comparison with other treatments, and determine whether other SGLT2 inhibitors, such as empagliflozin and canagliflozinare, can also improve the symptoms and prognosis of OSAHS patients.
